# A Static and Dynamic Combined Center of Mass Measurement Method Based on Multi-View Vision

**DOI:** 10.3390/s26165293

**Published:** 2026-08-21

**Authors:** Daojing Qu, Xuhao Zhang, Genyou Wei, Meibao Wang, Zhiyao Xiang

**Affiliations:** 1 School of Mechanical Engineering, Zhejiang Sci-Tech University, Hangzhou 310018, China; 17796048993@163.com (D.Q.); 15152454965@163.com (X.Z.); 19857125723@163.com (G.W.); 2 Zhejiang Zhongli Tools Manufacturing Co., Ltd., Lishui 321405, China; phdhit@126.com

**Keywords:** center of mass measurement, static and dynamic combined method, in situ measurement, binocular stereo vision

## Abstract

The position of the center of mass directly affects the attitude control and flight safety of moving bodies such as unmanned aerial vehicles (UAVs). Therefore, high-precision measurement of the center of mass is required. Existing methods require changing the posture of the measured object multiple times. This introduces repeated positioning errors and suffers from poor equipment versatility. To address these issues, this paper proposes a static and dynamic combined measurement method for the center of mass based on multi-view vision. First, the relationship between the swing period and the pendulum length under the simple pendulum principle is analyzed. The basic principle of determining the direction of the center of mass using the line of gravity is also examined. Second, an under-constrained compound pendulum fixture is designed. A binocular vision system is used to track circular markers, perform FFT-based period verification, and fit the gravity line using singular value decomposition (SVD). Third, using a standard cubic iron block as the test object, the influence of pendulum length and swing angle on measurement accuracy is studied. Finally, experiments verify that the proposed method can obtain three-dimensional coordinates of the center of mass under a single suspension condition. The results show that with a pendulum length of 330 mm and an initial swing angle of 4°, the root mean square error of the center of mass measurement is 0.70 mm, and the maximum deviation over five repeated measurements is 1.45 mm. This method does not require repeated lifting or changes in posture. It can meet the need for in-situ, high-precision center of mass measurement of UAVs and other aircraft.

## 1. Introduction

The center of mass is a core parameter describing the mass distribution of a rigid body. Its accurate determination directly affects the fidelity of dynamic modeling and the effectiveness of control algorithms. It is thus a key factor in ensuring system safety and mission accuracy. In engineering practice, whether for a DJI UAV inspecting complex environments or a small spacecraft in the design validation phase, a deviation in the center of mass can lead to attitude control failure, increased energy consumption, or even flight instability. Therefore, developing a high-precision, high-efficiency center of mass measurement technique suitable for field environments has important theoretical and engineering value for improving the dynamic performance and reliability of various moving bodies.

According to the measurement principle, existing experimental methods can be classified into static measurement, dynamic measurement, and frequency response function methods. A single static measurement cannot obtain three-dimensional coordinates of the center of mass. It requires changing the object’s posture at least twice, and each posture change introduces repeated positioning errors. Zhao et al. [1] systematically analyzed the random error factors in the three-point method for center of mass measurement. Modenini et al. [2] and Zhao et al. [3] analyzed the suspension method and the forward tilt method, respectively, but both require two or more measurements. Dynamic measurement methods also require multiple posture changes, and the equipment is often model-specific and expensive. The torsion pendulum method can measure moment of inertia [4,5] but still needs multiple posture changes. Yang et al. [6] used 3D point clouds, He et al. [7] combined 3D scanning with Hooke’s law, and Li et al. [8] proposed different methods for measuring the center of mass and moment of inertia based on multi-posture transformations. However, these methods still require multiple clamping operations. Gutierrez-Giles et al. [9] combined photogrammetry with electronic scales, Moemen et al. [10] used multi-mobile-robot machine vision, and Chu et al. [11,12] adopted either a multi-vision system combined with synthetic aperture imaging or a vision-based method for large components. All these rely on dedicated tooling or complex calibration. Lee and Kim [13] studied real-time center of mass estimation based on flight data, but the method is limited by model coupling. Similarly, Chen et al. [14] combined monocular vision with the torsion pendulum principle to measure moment of inertia, yet a dedicated torsion pendulum stand is still required. Zhang et al. [15] designed a general mass property measurement device for large aircraft, using multi-point supports and a force sensor array to jointly measure mass and center of mass, but multiple posture adjustments are still necessary. In recent years, emerging vision technologies such as active event cameras [16], visual SLAM algorithms [17], static-moving camera systems [18], and binocular stereo vision [19] have made significant progress in measurement. However, the application of such vision systems to center of mass measurement remains insufficiently explored. The frequency response function method can obtain all inertial parameters under a single excitation, but its measurement accuracy is low, and it usually ignores the stiffness and damping of the system, making it difficult to meet high-precision engineering requirements.

A review of the domestic and international literature shows that traditional mass property measurement techniques for the center of mass have become relatively mature and have formed a certain system. Nevertheless, they still face the following problems: long development cycles and high costs of measurement equipment, difficulty in covering objects with a wide range of sizes and masses, and poor equipment versatility [20]. In situ measurement has become a trend, but existing methods are mostly offline. Moreover, the improvement of measurement accuracy has hit a bottleneck. Traditional methods rely on high-precision sensors and ultra-precision mechanical structures, leaving little room for further accuracy enhancement.

To address the above problems and considering the practical engineering needs of UAVs, this paper proposes a static and dynamic combined center of mass measurement method based on multi-view vision. Compared with existing techniques, the main advantages of the proposed method are as follows. First, it determines the full three-dimensional coordinates of the center of mass under a single suspension condition, eliminating the repeated positioning errors inherent in multi-posture methods. Second, it combines static gravity line information with dynamic swing period measurement to obtain both the direction and the distance from the suspension point to the center of mass, enabling complete 3D localization from one experiment. Third, the use of a binocular vision system enables non-contact, real-time tracking of the swing motion without requiring object-specific tooling, making the method versatile and suitable for in situ applications. The method can also be used during the lifting process of large objects such as cars and aircraft, with simple and efficient equipment.

## 2. Basic Measurement Principle

**Figure 1:**
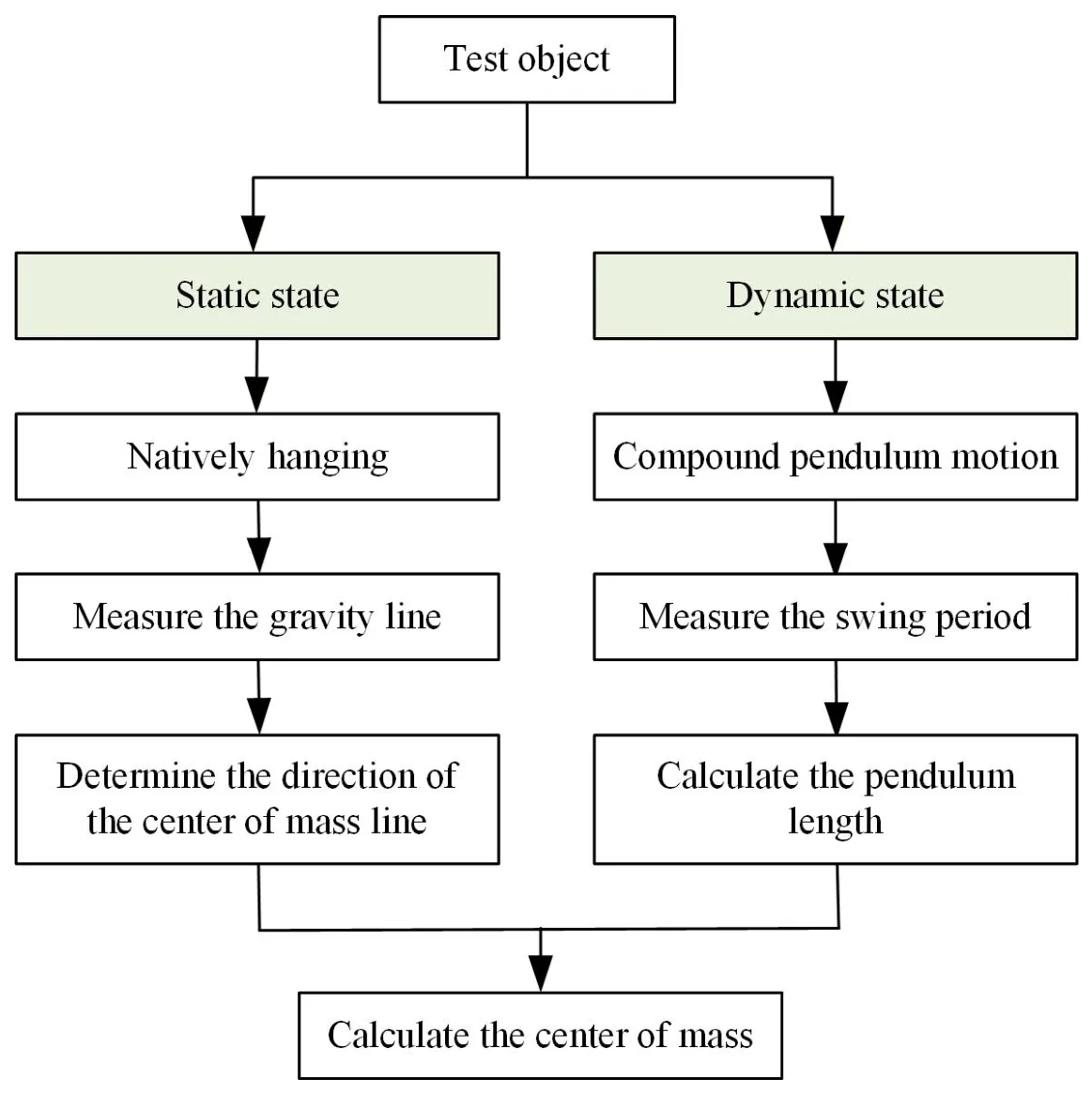
Block diagram of the center of mass measurement process.

The proposed calculation process for mass property parameters is shown in Figure 1. The object hangs naturally. When stationary, it hangs naturally under gravity, and the vertical line passing through the suspension point also passes through the object’s center of mass. The object can swing back and forth in a plane. By measuring the swing period, the distance from the suspension point to the center of mass (i.e., the pendulum length) is derived. At this point, both the direction and the distance from the suspension point to the center of mass are known, so the position of the center of mass can be determined. The object’s motion period, the direction of the gravity line, and the relationship with the object coordinate system are obtained using multi-view industrial cameras. Thus, by analyzing the object in two states (static and dynamic), the three-dimensional coordinates of the center of mass are obtained.

### 2.1. Determining the Gravity Line of the Center of Mass

**Figure 2:**
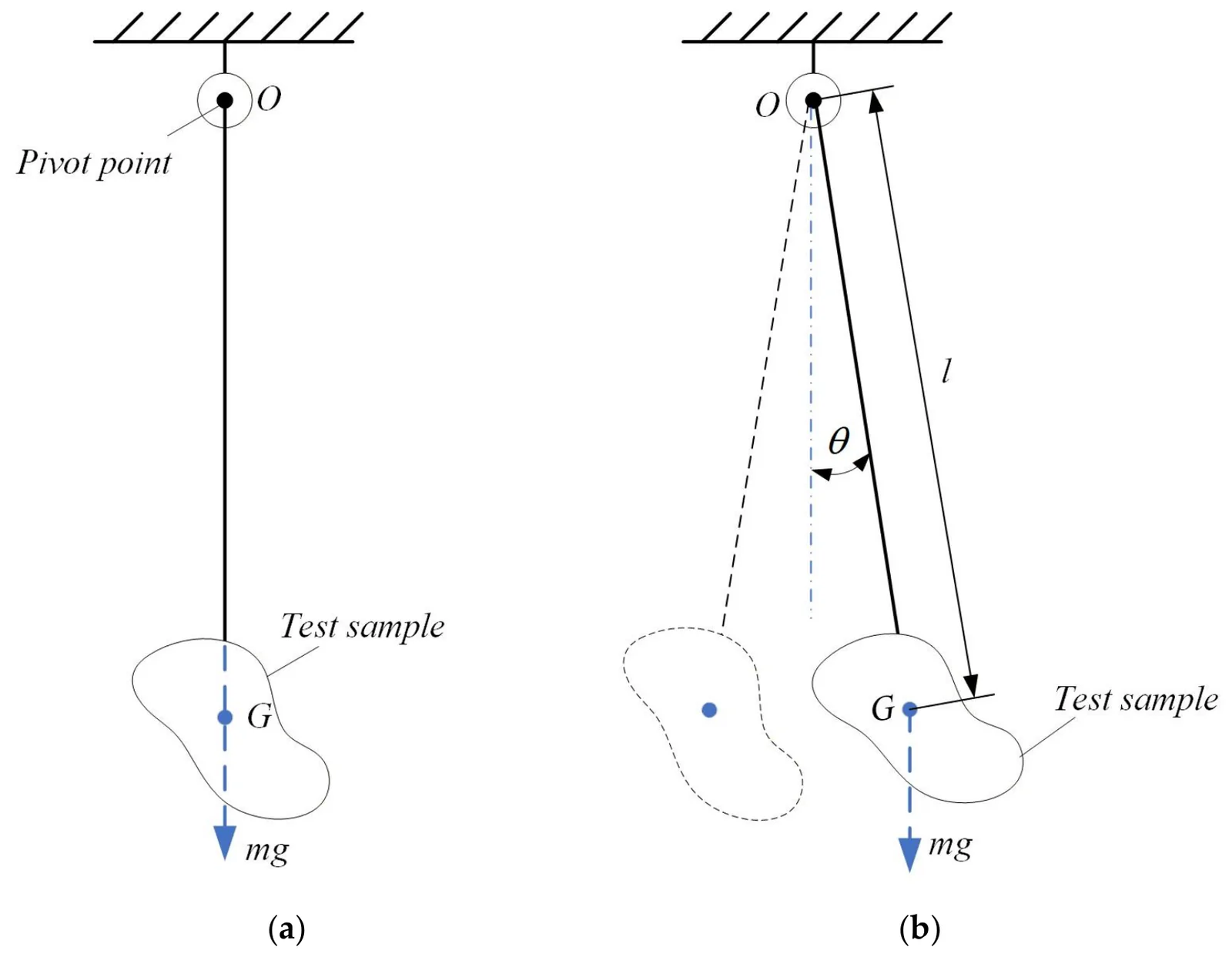
Schematic diagram of the measurement setup. (**a**) Static state: the object hangs naturally under gravity. The center of mass lies on the vertical line through the suspension point. (**b**) Compound pendulum motion: the object oscillates in a plane. The swing period is used to determine the pendulum length.

As shown in Figure 2a, the object is suspended by a long thin thread. It remains stationary after naturally hanging under gravity. The center of mass of the object lies on the line of the suspension thread.

Let $\left(x_{n},y_{n},z_{n}\right)$ be the coordinates of the n-th point on the gravity line in the measurement coordinate system, where n = 1, 2, 3, …. A straight line is fitted from these points. The general equation of the fitted line can be expressed as:

(1) $$\left\{\begin{array}{c} x=x_{0}+at\\ y=y_{0}+bt\\ z=z_{0}+ct \end{array}\right.$$

To simplify the calculation, Equation (1) can be written as:

(2) $$\frac{x-x_{0}}{a}=\frac{y-y_{0}}{b}=\frac{z-z_{0}}{c}$$

where $\left(x_{0},y_{0},z_{0}\right)$ are the coordinates of a known point on the line, t is a real parameter, and (a,b,c) is the direction vector of the gravity line.

A least-squares line fit is performed. Singular value decomposition (SVD) is used to find the optimal parameters $\left(x_{0},y_{0},z_{0},a,b,c\right)$ that minimize the error function defined as:

(3) $$E=\sum \left[{\left(x_{i}-x_{0}-at\right)}^{2}+{\left(y_{i}-y_{0}-bt\right)}^{2}+{\left(z_{i}-z_{0}-ct\right)}^{2}\right]$$

### 2.2. Determining the Pendulum Length

The fixture, the object, and the connecting parts are considered as a single rigid body system. As shown in Figure 2b, an excitation is applied to the object, causing it to oscillate back and forth around point O. The vibration equation of the compound pendulum is:

(4) $$J\frac{d^{2}\theta }{dt^{2}}+gml\sin\theta =0$$

where J is the moment of inertia of the whole rigid body system about the pivot axis O (unit: kg⋅m^2^), g is the gravitational acceleration (m/s^2^), m is the total mass of the rigid body system (kg), and l is the distance from the center of mass of the system to the pivot axis O (mm).

Neglecting air resistance is a common simplification in pendulum-based mass property measurements. As noted in the torsion pendulum literature, air damping can be neglected when measuring general axisymmetric bodies [21]. For small-sized and simply shaped objects, the influence of air damping is negligibly small in practical measurements; it becomes appreciable only for large complex structures with large windward areas [22]. For a lightly damped pendulum with small oscillation amplitude, the energy dissipated by air resistance per cycle is proportional to the square of the velocity and is of second order relative to the potential energy variation. Therefore, when the initial swing angle θ_0_ is very small (θ_0_≪5°), the air resistance term in Equation (4) can be omitted, yielding the simplified form:

(5) $$J\frac{d^{2}\theta }{dt^{2}}+gml\theta =0$$

The swing period T is given by:

(6) $$T=2\pi \sqrt{\frac{J}{mgl}}$$

Using the parallel axis theorem, the moment of inertia J about the pivot axis O can be expressed as:

(7) $$J=J_{c}+ml^{2}$$

where J_C_ is the moment of inertia of the system about its own center of mass. Substituting (7) into (6) gives:

(8) $$T=2\pi \sqrt{\frac{J_{c}+ml^{2}}{mgl}}$$

When the system satisfies J_C_ ≪ ml^2^, the term J_C_ in Equation (8) can be neglected, leading to the simple pendulum formula:

(9) $$T=2\pi \sqrt{\frac{l}{g}}$$

From Equation (9), the distance l from the center of mass to the pivot point is calculated as:

(10) $$l=\frac{T^{2}g}{4\pi^{2}}$$

We define the simplification factor κ as:

(11) $$\kappa =\frac{J_{c}}{ml^{2}}$$

Equation (10) holds when κ ≪ 1.

### 2.3. Determining the Center of Mass

The pendulum length l is obtained from Equation (10), and the direction vector (a,b,c) of the gravity line is obtained from Equation (2). Let the coordinates of the center of mass G be (x,y,z). Then G lies on the line expressed by Equation (2), and the distance from G to the pivot axis O is l. That is:

(12) $$x^{2}+y^{2}+z^{2}=l$$

Substituting Equation (2) into (12) yields:

(13) $${\left(x_{0}+at\right)}^{2}+{\left(y_{0}+bt\right)}^{2}+{\left(z_{0}+ct\right)}^{2}=l$$

Thus t can be solved, and substituting it back into Equation (2) gives the coordinates G=(x,y,z) of the center of mass.

## 3. Key Technologies

### 3.1. Circular Marker Detection and Tracking

To achieve non-contact measurement of the swing trajectory and the gravity line, a binocular vision system is used to detect and track circular markers on the fixture. The markers are red circles. They are recognized by contour detection and HSV colour space filtering. For each candidate contour, the area, perimeter, and circularity are calculated to filter out the circular markers. During tracking, each marker is assigned a fixed ID, and independent tracking of multiple markers is achieved by matching the smallest Euclidean distance between frames.

The pixel coordinates (u,v) of a marker are obtained, and its depth value d (in meters) is acquired from a depth camera. Using the camera intrinsic parameters, the pixel coordinates are transformed to three-dimensional coordinates $\left(x_{c},y_{c},z_{c}\right)$ in the camera coordinate system:

(14) $$\left\{\begin{array}{c} x_{c\;=\;}\frac{(u-c_{x})\cdot d}{f_{x}}\\ y_{c}=\frac{(v-c_{y})\cdot d}{f_{y}}\\ z_{c}=d \end{array}\right.$$

where $\left(c_{x},c_{y}\right)$ are the principal point coordinates (in pixels) and $\left(f_{x},f_{y}\right)$ are the focal lengths in pixels.

### 3.2. Measurement and Extraction of the Compound Pendulum Period

To obtain the swing period T in Equation (10), the camera records the motion sequence of feature points at a certain sampling frequency. The horizontal position sequence x(t) of a single marker is analyzed. First, a moving average filter is applied to smooth high-frequency noise. Then peak detection is used to locate the wave peaks. The time differences between adjacent peaks are averaged after removing outliers to obtain the swing period T. At the same time, a fast Fourier transform (FFT) is used to verify the period. FFT is a fast algorithm that converts a time-domain discrete signal into a frequency-domain representation. Its core is to reduce the computational complexity of the discrete Fourier transform (DFT) from $O\left(N^{2}\right)$ to $O\left(NlogN\right)$ using a divide-and-conquer strategy, greatly improving spectral analysis efficiency.

In this system, the mean value is subtracted from the marker’s horizontal position sequence x[n](n = 0, 1, …, N − 1) to remove the DC component. Then the FFT is performed as:

(15) $$X[k]=\sum_{n=0}^{N-1}x[n]\cdot e^{-j\cdot 2\pi \mathrm{kn}/N}(k\;=\;0,\;1,\;\ldots ,N-1)$$

After obtaining the spectrum X[k], the frequency index k_max_ corresponding to the maximum amplitude ∣X[k]∣ is found. Under the small-angle linear approximation, the main frequency is:

(16) $$f_{0}=\frac{k_{\max}\cdot f_{s}}{N}$$

where f_s_ is the sampling frequency. The corresponding period T is:

(17) $$T=\frac{1}{f_{0}}$$

Under the experimental conditions, the Nyquist condition (f_s_ > 2 times the swing frequency) is satisfied. Physically, periodic swinging in the time domain appears as a single prominent spectral peak in the frequency domain. Extracting the frequency of this peak gives the fundamental frequency of the swing. The FFT method is based on the global spectral characteristics of the entire signal, complementing the peak detection method (which is a local extremum approach): the former is more robust to noise, while the latter offers better intuitive interpretability of the signal waveform.

### 3.3. Measuring the Gravity Line

Assume there are m circular markers on the fixture, with coordinates $p_{i}={\left(x_{i},y_{i},z_{i}\right)}^{T}$ in the camera coordinate system, i = 1, …, m. Construct the matrix $P\in R^{m\times 3}$, where the i-th row is $p_{i}^{T}$. We compute the mean vector:

(18) $$\mu =\frac{1}{m}\sum_{i=1}^{m}p_{i}$$

We center the data:

(19) $$\bar{P}=P-1\mu^{T}$$

We perform singular value decomposition (SVD) on $\bar{P}$

(20) $$\bar{P}=U\sum V^{T}$$

where $V=[v_{1},v_{2},v_{3}]$ is the right singular matrix. The right singular vector v_1_ corresponding to the largest singular value σ_1_ is the principal direction of the data point distribution, i.e., the direction vector ${\left(a,b,c\right)}^{T}$ of the gravity line. This direction minimizes the sum of squared orthogonal distances from all points to the line. Here, the SVD fit directly gives the direction vector of the line in Equation (1), avoiding the explicit minimization of the error function in Equation (3); the two are mathematically equivalent.

The geometric interpretation of SVD-based line fitting is as follows. For a set of 3D points $p_{i}$ that are approximately collinear, the centroid $\bar{P}$ is first subtracted to obtain the centered data matrix $P\in R^{m\times 3}$. SVD decomposes $\bar{P}$ as $\bar{P}=U\sum V^{T}$, where $V=[v_{1},v_{2},v_{3}]$ contains the right singular vectors and ∑=diag(σ1,σ2,σ3), with σ1≥σ2≥σ3≥0. The first right singular vector v_1_, corresponding to the largest singular value σ_1_, represents the direction of maximum variance of the point set, namely the direction of the fitted line. The fitted line passes through the centroid $\bar{P}$ and has direction v_1_. This is equivalent to minimizing the sum of squared orthogonal distances from all points to the line, i.e., three-dimensional orthogonal distance regression. In addition, the ratios σ_2_/σ_1_ and σ_3_/σ_1_ can be used to evaluate the collinearity of the marker points; values close to zero indicate that the points are well aligned along a straight line.

Applying this principle to gravity line measurement: when the object hangs naturally, the multiple circular markers on the plumb line of the fixture align along the vertical direction due to gravity. The principal direction of their spatial distribution necessarily coincides with the gravity direction. Therefore, after performing SVD on the marker coordinate matrix, the first principal component direction is the direction vector (a,b,c) of the gravity line. The direction vector is normalized after fitting. If the direction points upward (i.e., its dot product with the positive Y-axis is positive), it is automatically reversed to ensure that the gravity direction points toward the center of the Earth.

### 3.4. Center of Mass Results and Coordinate Transformation

From Section 2.3, the coordinates of the center of mass in the camera coordinate system are obtained as $C_{cam}={\left(x,y,z\right)}^{T}$. To express the center of mass position in the object’s own coordinate system, a transformation relationship between the object coordinate system and the camera coordinate system must be established.

For a standard part with known geometry (or for a test object with three non-collinear markers), three markers are selected: point A (defined as the origin of the object coordinate system), point B (used to define the X-axis direction), and point C (lying in the XY plane). Their coordinates in the camera coordinate system are A, B, and C respectively.

First, we calculate the Z-axis direction vector and normalize it:

(21) $$Z=\frac{A-B}{\left\|A-B\right\|}$$

Then we compute the X-axis direction by crossing $\vec{AC}$ with $\vec{BA}$:

(22) $$X=\frac{\left(A-B)\times (C-A\right)}{\left\|\left(A-B)\times (C-A\right)\right\|}$$

Finally, complete the right-handed orthogonal system by obtaining the Y-axis as the cross product of Z and X:

(23) Y=Z×X

Form the rotation matrix R by taking the three basis vectors as column vectors. This matrix transforms coordinates from the object coordinate system to the camera coordinate system:

(24) $$R=[XYZ]\in R^{3\times 3}$$

Since R is orthogonal ($RR^{T}=I$), its inverse equals its transpose:

(25) $$R^{-1}=R^{T}$$

For any point P_camera_ in the camera coordinate system, its coordinates P_object_ in the object coordinate system are obtained by translation and rotation:

(26) $$P_{object}=R^{T}(P_{\mathrm{camera}}-A)$$

where A is the position of the origin in the camera coordinate system.

We introduce a 4 × 4 homogeneous transformation matrix. We define the homogeneous transformation matrix from the camera coordinate system to the object coordinate system as:

(27) $$T_{cam\to obj}=\left[\begin{array}{cc} R^{T} & -R^{T}A\\ 0^{T} & 1 \end{array}\right]$$

Then the transformation in homogeneous coordinates is:

(28) $$\left[\frac{P_{object}}{1}\right]=T_{cam\to obj}\left[\frac{P_{\mathrm{camera}}}{1}\right]$$

## 4. Experimental Tests

### 4.1. Experimental Platform and Object

**Figure 3:**
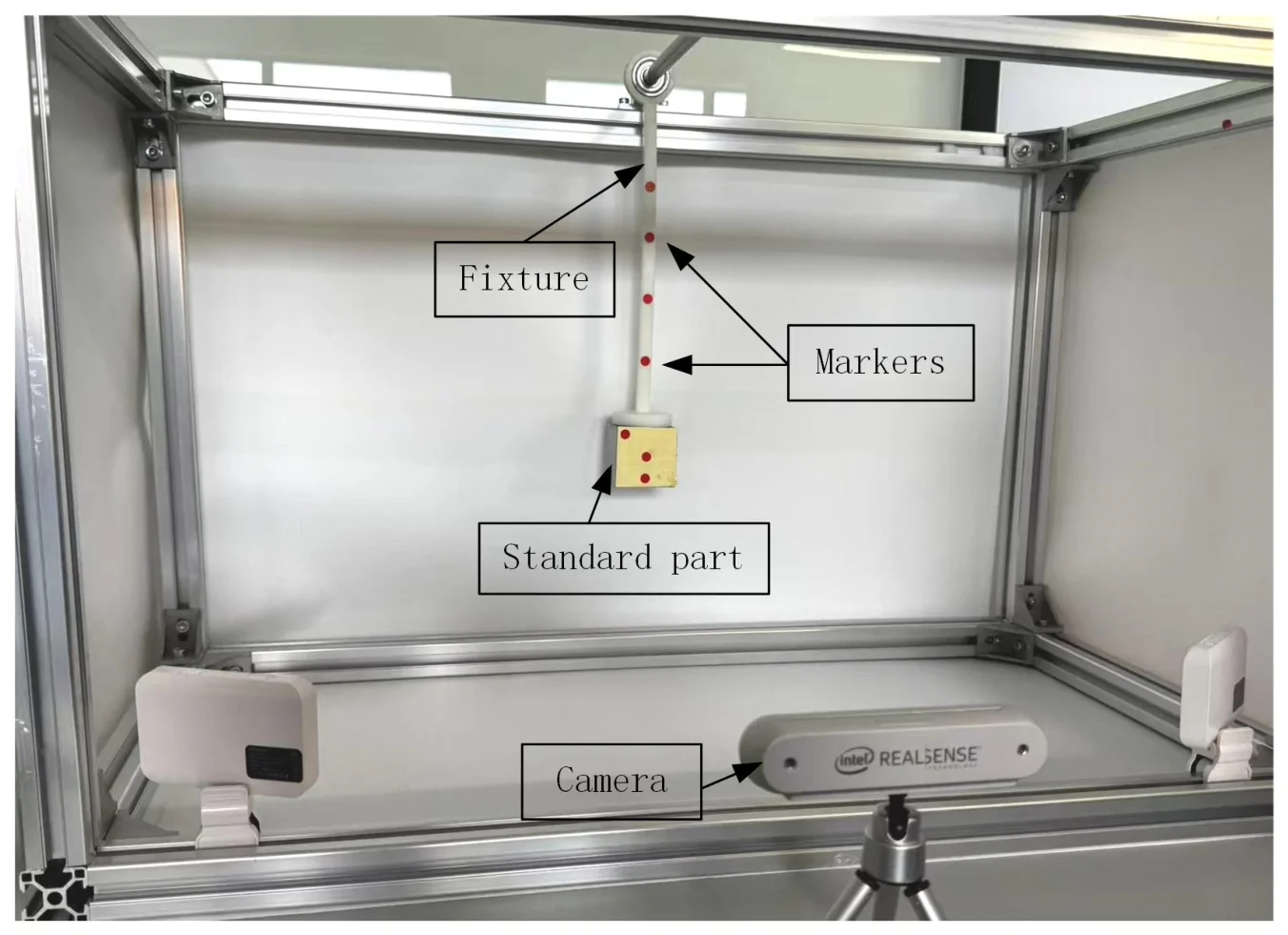
Standard part center of mass measurement platform. The platform consists of the compound pendulum fixture, circular markers, the standard cubic iron block, and the binocular vision system (Intel RealSense cameras).

As shown in Figure 3, the experimental platform consists of a binocular vision system composed of an Intel RealSense D455 depth camera (Intel Corporation, Santa Clara, CA, USA) and a self-designed compound pendulum fixture. The camera resolution is 1280 × 720 pixels, and the acquisition frame rate is 30 fps. Circular reflective markers are attached to the fixture for visual tracking. Image acquisition and processing were implemented using the Intel RealSense SDK 2.0 with Python 3.8, OpenCV 4.5, and NumPy 1.21.

Lighting conditions. Since the circular markers are detected using HSV colour space filtering, the ambient lighting can affect the robustness of marker segmentation. To ensure reliable marker detection, two external light sources were positioned symmetrically on both sides of the pendulum fixture to provide uniform illumination on the marker surfaces. Under this lighting configuration, the red circular markers exhibit sufficient contrast against the background, and the HSV filtering thresholds remain stable across the measurement sequence.

The test object is a standard cubic iron block with a side length of 50 mm and a mass of 980 g. Due to its uniform density and precise machining, the theoretical center of mass is at the geometric center, i.e., (0, 0, 25) mm in the object coordinate system. To study the influence of the pendulum length on measurement accuracy, three sets of fixtures were designed with pendulum lengths of approximately 330 mm (Fixture 1, mass 42.5 g), 310 mm (Fixture 2, mass 39 g), and 290 mm (Fixture 3, mass 37 g). Each fixture is a slender, lightweight rod structure designed to minimize its own moment of inertia about the center of mass, thereby ensuring that the system approximates a simple pendulum.

The measurement procedure consists of the following steps:

(1) Fixture preparation. The standard cubic iron block is securely mounted to the compound pendulum fixture. Three circular reflective markers are attached along the plumb line of the fixture.
(2) Static acquisition. The object is allowed to hang naturally until it comes to rest. The camera captures 50 frames of the static markers. The 3D coordinates of each marker are averaged over the 50 frames to reduce random noise.
(3) Dynamic acquisition. A small initial displacement (θ_0_ = 4° for the optimal case) is applied to the object, and the camera records the swinging motion for 20 complete periods.
(4) Real-time data processing. The image streams are processed in real time. The swing period T is extracted via both peak detection and FFT verification. The gravity line is fitted using SVD. The center of mass coordinates are then calculated using Equations (10)–(13) and displayed immediately after each measurement.
(5) Repeatability assessment. The above procedure is repeated five times under identical conditions to assess repeatability.

### 4.2. Center of Mass Measurement of the Standard Part

To verify the measurement accuracy of the proposed method, a cubic iron block with side length 50 mm was used as the standard part (uniform density, theoretical center of mass at the geometric center, (0, 0, 25) mm in the object coordinate system). Three fixtures with different pendulum lengths were designed: Fixture 1 (≈330 mm), Fixture 2 (310 mm), and Fixture 3 (290 mm). Before each measurement, the object coordinate system was defined using three known markers, and the calculated center of mass was transformed to the object coordinate system. Three sets of experiments were conducted. Experiment 1: Pendulum length comparison—Measurements were repeated using Fixtures 1, 2, and 3 to evaluate the effect of pendulum length on accuracy. Experiment 2: Swing angle comparison—Fixture 1 was used under different initial swing angles. Experiment 3: Repeatability test—Fixture 1 was used under the optimal conditions for five repeated measurements. The results are shown in Table 1, Table 2 and Table 3. The detailed raw data for all three experiments are provided in Appendix A (Table A1, Table A2 and Table A3).

As shown in Table 1, a longer pendulum length leads to higher center of mass measurement accuracy. The RMS error of Fixture 1 is 0.70 mm, while that of Fixture 3 is 3.83 mm.

As shown in Table 2, when the swing angle is 4°, the measurement error is smallest (RMS = 0.70 mm). When the angle increases to 10°, the RMS increases to 3.91 mm. At 17°, the RMS reaches 10.24 mm. This is because a small-angle swing is closer to the undamped small-angle assumption of the simple pendulum (sin θ≈θ). As the angle increases, nonlinear effects become stronger. Therefore, the initial swing angle should be kept within 4° in practical measurements.

Under the optimal conditions (Fixture 1, swing angle 4°), five repeated measurements were performed, and the results are shown in Table 3. The maximum RMS error is 1.45 mm, indicating good repeatability of the proposed method.

## 5. Error Analysis

(1) Model simplification error. In practice, the actual system is usually a compound pendulum, as described by Equation (4). This paper first applied the small-angle approximation to simplify Equation (4). Therefore, the method is applicable when the initial swing angle θ_0_ is very small (θ_0_ ≪ 1 rad). Furthermore, the compound pendulum model is further simplified using the simple pendulum formula. The simplification is valid when η ≪ 1 in Equation (11). By designing a slender, lightweight fixture and ensuring that the suspension point is far from the system’s center of mass, this condition can be satisfied, thus keeping the model simplification error within an acceptable range.
(2) Vision measurement error. The pixel error and depth noise when the binocular vision system extracts the three-dimensional coordinates of markers are the main sources of random error. These errors can be effectively suppressed by sub-pixel extraction, multi-frame averaging, increasing the number of plumb-line markers, and enlarging their distribution span. Under the experimental conditions described in this paper, the calibrated vision positioning accuracy is better than 0.2 mm. Further accuracy improvement can be achieved by using higher-resolution cameras, better depth sensing modules, and systematic calibration compensation.
(3) Period extraction error. The camera sampling frequency used in this paper is 30 Hz, which introduces temporal quantization error in a single-period measurement. By averaging multiple periods and using FFT spectral verification, the relative error of the period measurement can be controlled within 0.1%. Using a camera with a higher sampling frequency can further reduce this error.

## 6. Conclusions

To address the problems of existing center of mass measurement methods—namely, the need for multiple changes of the object’s posture, the introduction of repeated positioning errors, and poor equipment versatility—this paper proposed a static and dynamic combined center of mass measurement method based on multi-view vision. First, the basic principle of using a compound pendulum to measure the swing period under a single suspension, calculating the pendulum length, and then determining the three-dimensional coordinates of the center of mass together with the gravity line was introduced. The influence of factors such as pendulum length and swing angle on measurement accuracy was analyzed. Then, the implementation methods of key technologies in the multi-view vision system were described, including circular marker detection, FFT-based swing period verification, SVD fitting of the gravity line, and coordinate system transformation. Finally, an experimental platform was built using a standard cubic iron block for validation. The experimental results show that under the optimal conditions (pendulum length ≈ 330 mm, initial swing angle 4°), the root mean square error of the center of mass measurement for the standard part is 0.70 mm, and the maximum deviation over five repeated measurements is 1.45 mm. The method does not require repeated lifting or changing the object’s posture. It can meet the needs of in situ, high-precision center of mass measurement for UAVs and other aircraft.

## Figures and Tables

**Table 1:** Center of mass measurement results for the standard part with different fixtures (object coordinate system).

Fixture	Measured Center of Mass (mm)	Error Vector (mm)	RMS (mm)
Fixture 1	(0.10, 1.03, 24.38)	(0.10, 1.03, −0.62)	0.70
Fixture 2	(2.49, 0.68, 26.40)	(2.49, 0.68, 1.40)	1.69
Fixture 3	(6.44, 1.26, 26.00)	(6.44, 1.26, 1.00)	3.83

**Table 2:** Center of mass measurement results for the standard part with Fixture 1 at different swing angles (object coordinate system).

Swing Angle	Measured Center of Mass (mm)	Error Vector (mm)	RMS (mm)
4°	(0.10, 1.03, 24.38)	(0.10, 1.03, −0.62)	0.70
10°	(6.70, 0.75, 24.43)	(6.70, 0.75, −0.57)	3.91
17°	(7.51, −15.95, 23.00)	(7.51, −15.945, −2.00)	10.24

**Table 3:** Center of mass measurement results for the standard part with Fixture 1 at a small swing angle (object coordinate system).

Measurement Set	Measured Center of Mass (mm)	Error Vector (mm)	RMS (mm)
Set 1	(−0.24, −0.69, 24.415)	(−0.24, −0.69, −0.68)	0.54
Set 2	(0.10, 1.03, 24.38)	(0.10, 1.03, –0.62)	0.70
Set 3	(0.80, −1.30, 27.00)	(0.80, −1.30, 2.00)	1.45
Set 4	(1.89, −1.36, 24.96)	(1.89, −1.35, −0.04)	1.34
Set 5	(1.93, 1.05, 24.91)	(1.93, 1.05, −0.09)	1.27

## Data Availability

The raw data presented in this study are available in Appendix A of this article.

## Appendix A. Raw Data Tables

**Table A1:** Raw data for the three fixture configurations (Experiment 1: Pendulum length comparison).

Fixture	Period (s)	Pendulum (mm)	Reference Point of the Object Coordinate System
Fixture 1	1.167	338.4	ID0 (12.4, 47.5, 379.0) ID1 (−7.2, 9.2, 380.0) ID2 (12.4, 27.9, 280.0)
Fixture 2	1.130	317.1	ID0 (22.0, 76.3, 376.0) ID1 (21.9, 58.6, 375.0) ID2 (4.5, 39.0, 374.0)
Fixture 3	1.101	301.4	ID0 (2.5, 48.1, 401.0) ID1 (−3.5, 65.8, 401.0) ID2 (21.1, 29.4, 401.0)

**Table A2:** Raw data for different swing angles using Fixture 1 (Experiment 2: Swing angle comparison).

Fixture	Period (s)	Pendulum (mm)	Reference Point of the Object Coordinate System
4°	1.167	338.4	ID0 (12.4, 47.5, 379.0) ID1 (−7.2, 9.2, 380.0) ID2 (12.4, 27.9, 280.0)
10°	1.171	341.0	ID0 (1.6, 91.5, 391.0) ID1 (17.6, 73.2, 391.0) ID2 (1.6, 111.6, 390.0)
17°	1.176	343.8	ID0 (11.0, 54.4, 367.0) ID1 (−7.9, 36.4, 367.0) ID2 (12.0, 74.4, 367.0)

**Table A3:** Raw data for five repeated measurements under optimal conditions (Experiment 3: Repeatability test).

Fixture	Period (s)	Pendulum (mm)	Reference Point of the Object Coordinate System
Set 1	1.167	338.4	ID0 (12.4, 47.5, 379.0) ID1 (−7.2, 9.2, 380.0) ID2 (12.4, 27.9, 280.0)
Set 2	1.167	339.5	ID0 (16.8, 45.2, 369.0) ID1 (16.8, 63.4, 369.0) ID2 (−1.3, 26.0, 368.0)
Set 3	1.167	338.1	ID0 (−6.1, 46.7, 373.0) ID1 (−6.1, 27.4, 373.0) ID2 (−25.4, 9.0, 373.0)
Set 4	1.169	339.4	ID0 (18.4, 27.0, 369.0) ID1 (−38.8, 8.9, 368.0) ID2 (18.5, 47.1, 369.0)
Set 5	1.167	338.2	ID0 (−37.5, 8.9, 369.0) ID1 (18.4, 27.0, 369.0) ID2 (18.5, 47.2, 370.0)
